# Systematic review of statistics on causes of deaths in hospitals: strengthening the evidence for policy-makers

**DOI:** 10.2471/BLT.14.137935

**Published:** 2014-09-16

**Authors:** Rasika Rampatige, Lene Mikkelsen, Bernardo Hernandez, Ian Riley, Alan D Lopez

**Affiliations:** a University of Queensland, School of Population Health, Brisbane, Australia.; b LM Consulting, Brisbane, Australia.; c Institute for Health Metrics and Evaluation, University of Washington, Seattle, United States of America.; d University of Melbourne, Melbourne School of Population and Global Health, Building 379, 207 Bouverie Street, Carlton 3053, Victoria, Australia.

## Abstract

**Objective:**

To systematically review the reliability of hospital data on cause of death and encourage periodic reviews of these data using a standard method.

**Methods:**

We searched Google Scholar, Pubmed and Biblioteca Virtual de la Salud for articles in English, Spanish and Portuguese that reported validation studies of data on cause of death. We analysed the results of 199 studies that had used medical record reviews to validate the cause of death reported on death certificates or by the vital registration system.

**Findings:**

The screened studies had been published between 1983 and 2013 and their results had been reported in English (*n* = 124), Portuguese (*n* = 25) or Spanish (*n* = 50). Only 29 of the studies met our inclusion criteria. Of these, 13 had examined cause of death patterns at the population level – with a view to correcting cause-specific mortality fractions – while the other 16 had been undertaken to identify discrepancies in the diagnosis for specific diseases before and after medical record review. Most of the selected studies reported substantial misdiagnosis of causes of death in hospitals. There was wide variation in study methodologies. Many studies did not describe the methods used in sufficient detail to be able to assess the reproducibility or comparability of their results.

**Conclusion:**

The assumption that causes of death are being accurately reported in hospitals is unfounded. To improve the reliability and usefulness of reported causes of death, national governments should do periodic medical record reviews to validate the quality of their hospital cause of death data, using a standard.

## Résumé

**Objectif:**

Examiner systématiquement la fiabilité des données hospitalières sur la cause des décès et encourager l'examen périodique de ces données en utilisant une méthode standard.

**Méthodes:**

Nous avons utilisé Google Scholar, Pubmed et Biblioteca Virtual de la Salud pour rechercher des articles en anglais, en espagnol et en portugais, qui ont rapporté des études de validation des données sur la cause des décès. Nous avons analysé les résultats de 199 études qui ont utilisé des examens de dossiers médicaux pour valider la cause de la mort déclarée sur les certificats de décès ou par le système d'enregistrement de l'état civil.

**Résultats:**

Les études passées au crible ont été publiées entre 1983 et 2013, et leurs résultats ont été publiés en anglais (*n* = 124), en portugais (*n* = 25) ou en espagnol (*n* = 50). Seules 29 études ont rempli nos critères d'inclusion. Parmi ces études, 13 ont étudié les schémas des causes de décès au niveau de la population – en vue de corriger les fractions de mortalité selon l'âge – alors que les 16 autres ont été entreprises pour identifier les contradictions dans le diagnostic des maladies spécifiques, avant et après l'examen du dossier médical. La plupart des études sélectionnées ont rapporté des erreurs importantes de diagnostic pour les causes de décès dans les hôpitaux. Il y avait une grande variation dans les méthodologies d'étude. De nombreuses études ne décrivaient pas suffisamment les méthodes utilisées pour pouvoir évaluer la reproductibilité ou la comparabilité de leurs résultats.

**Conclusion:**

L'hypothèse selon laquelle les causes de décès sont déclarées de manière précise dans les hôpitaux est sans fondement. Pour améliorer la fiabilité et l'utilité des causes de décès déclarées, les gouvernements nationaux devraient effectuer des examens périodiques des dossiers médicaux afin de valider la qualité des données sur la cause des décès dans leurs hôpitaux, en utilisant une méthode standard permettant d'obtenir des résultats comparables au niveau international.

## Resumen

**Objetivo:**

Revisar sistemáticamente la fiabilidad de los datos de los hospitales sobre las causas de las muertes y promover revisiones periódicas de estos datos por medio de un método estándar.

**Métodos:**

Se realizaron búsquedas de artículos en inglés, español y portugués en Google Scholar, Pubmed y la Biblioteca Virtual de la Salud sobre estudios que informaran acerca de la validación de datos sobre las causas de las muertes. Analizamos los resultados de 199 estudios que habían empleado revisiones de registros médicos para validar las causas de las muertes registradas en los certificados de defunción o por el sistema de registro civil.

**Resultados:**

Los estudios seleccionados se habían publicado entre 1983 y 2013 y sus resultados aparecían en inglés (n = 124), portugués (n = 25) o español (n = 50). Solo 29 de los estudios cumplieron los criterios de inclusión. De estos, 13 habían examinado las causas de muerte en la población con el fin de corregir las fracciones de mortalidad por una causa concreta, mientras que los otros 16 pretendían identificar las discrepancias en el diagnóstico de enfermedades específicas antes y después de la revisión de los historiales médicos. La mayoría de los estudios seleccionados informaron acerca de diagnósticos sustancialmente incorrectos de las causas de muerte en los hospitales. Las metodologías de estudio mostraron una amplia variación. Muchos estudios no describieron los métodos utilizados de manera suficientemente detallada como para poder evaluar la reproducibilidad o la comparabilidad de sus resultados.

**Conclusión:**

La suposición de que los hospitales notifican adecuadamente las causas de la muerte es infundada. A fin de mejorar la fiabilidad y la utilidad de las causas de muerte notificadas, los gobiernos nacionales deberían llevar a cabo revisiones periódicas de los historiales médicos con objeto de validar la calidad de los datos hospitalarios sobre las causas de muertes por medio de un método estándar para lograr resultados comparables a nivel internacional.

## ملخص

الغرض إجراء استعراض منهجي لموثوقية بيانات المستشفيات الخاصة بسبب الوفاة وتشجيع إجراء استعراضات دورية لهذه البيانات باستخدام طريقة موحدة.

الطريقة تم إجراء بحث في Google Scholar وPubmed وBiblioteca Virtual de la Salud للحصول على الموضوعات المكتوبة باللغات الإنجليزية والإسبانية والبرتغالية التي أبلغت عن دراسات تحقق للبيانات بشأن سبب الوفاة. وقمنا بتحليل نتائج 199 دراسة استخدمت استعراضات السجل الطبي للتحقق من سبب الوفاة المبلغ عنه في شهادات الوفاة أو عن طريق نظام تسجيل الأحوال المدنية.

النتائج تم نشر الدراسات التي تم فحصها بين 1983 و2013 وتم الإبلاغ عن نتائجها باللغة الإنجليزية (العدد = 124) أو البرتغالية (العدد = 25) أو الإسبانية (العدد = 50). ولم تستوف سوى 29 دراسة معايير الإدراج الخاصة بنا. وتناولت 13 دراسة من هذه الدراسات سبب أنماط الوفاة على الصعيد السكاني - بغية تصحيح نسب معدل الوفيات المحدد بالأسباب - في حين تم إجراء الست عشرة دراسة الأخرى لتحديد أوجه التباين في تشخيص أمراض معينة قبل استعراض السجل الطبي وبعده. وأبلغت معظم الدراسات المحددة عن تشخيص خاطئ على نحو كبير لأسباب الوفاة في المستشفيات. وكان هناك تفاوت كبير في منهجيات الدراسة. ولم تصف العديد من الدراسات الطرق المستخدمة بتفصيل كاف يتسنى معه تقييم قابلية تكرار نتائجها أو مقارنتها.

الاستنتاج لا يوجد أساس لافتراض دقة الإبلاغ عن أسباب الوفاة في المستشفيات. ولتحسين موثوقية أسباب الوفاة المبلغ عنها وفائدتها، ينبغي أن تجري الحكومات الوطنية استعراضات دورية للسجلات الطبية للتحقق من جودة سبب بيانات الوفاة الذي يتم الإبلاغ به بواسطة المستشفيات، باستخدام طريقة موحدة لتحقيق نتائج يمكن مقارنتها على الصعيد الدولي.

## 摘要

**目的:**

系统回顾医院死因数据的可靠性并鼓励使用标准方法定期回顾这些数据。

**方法:**

我们搜索Google Scholar、Pubmed和Biblioteca Virtual de la Salud中报告死因数据验证研究的英语、西班牙语和葡萄牙语文章。我们分析了199个研究的结果,这些研究利用医疗记录回顾来验证死亡证明报告的死因或由生命登记系统报告的死因。

**结果:**

筛选的研究发表在1983年和2013年之间,其研究结果以英语(n = 124)、葡萄牙语(n = 25)和西班牙语(n = 50)发表。只有29项研究满足我们的入选标准。这其中,13项研究在人口水平调查死因模式(旨在纠正死因别死亡率分数),而另外16项识别特定疾病的诊断在病历回顾前后的差异。大多数的所选择的研究报道了大量对医院死亡原因的错误诊断。研究方法存在很大差异。许多研究未详细描述所用的方法,不足以评估结果的再现性和可比性。

**结论:**

医院准确报道死因的假设是没有根据的。为改善死因报告的可靠性和实用性,各国政府应该使用标准方法完成定期的病历回顾来验证医院死因数据的质量,以取得国际可比性的结果。

## Резюме

**Цель:**

Провести систематический обзор достоверности больничных данных о причинах смерти и способствовать проведению периодических обзоров таких данных, используя стандартный метод.

**Методы:**

Был выполнен поиск статей на порталах Google Scholar, PubMed и Biblioteca Virtual de la Salud, написанных на английском, испанском и португальском языках, в которых приводились проверочные исследования данных о причинах смерти. Были проанализированы результаты 199 исследований, в которых были проведены обзоры медицинских записей для подтверждения причин смерти, указанных в свидетельствах о смерти или в записях актов гражданского состояния.

**Результаты:**

Отобранные исследования были опубликованы в 1983-2013 гг. и их результаты были представлены на английском (*n* = 124), португальском (*n* = 25) или испанском (*n* = 50) языках. Только 29 из этих исследований соответствовали критериям включения. Из них в 13 исследованиях рассматривались причины смертей на популяционном уровне — с целью коррекции долей конкретных причин смертности, а еще 16 были проведены для выявления расхождений в диагностике конкретных заболеваний до и после проверки медицинской документации. В большинстве из выбранных исследований сообщалось о значительных ошибках в установлении причин смерти в больницах. Методологии исследований значительно различались между собой. Многие исследования не содержали достаточно подробного описания используемых методов, чтобы можно было оценить воспроизводимость или сопоставимость их результатов.

**Вывод:**

Предположение, что в больницах ведется точная отчетность о причинах смерти, является необоснованным. Для повышения достоверности и полезности данных о сообщаемых причинах смерти национальные правительства должны проводить периодические обзоры медицинских записей с целью проверки качества данных о причинах смерти, предоставляемых больницами, используя стандартный метод для достижения международной сопоставимости результатов.

## Introduction

The poor state of health information systems and, particularly, mortality statistics in many countries is widely documented in the literature and in country reports to the World Health Organization (WHO).^1^^–^^5^ However, health systems worldwide depend on reliable information about causes of mortality to be able to respond effectively to changing epidemiological circumstances. Such responses depend critically on accurate data to guide decision-making.^6^ Within a health information system, accurate and timely data on the cause of death are fundamental for programme and policy development^7^ and for measuring change in the magnitude and distribution of ill-health and disease in populations.^8^ Assessments of vital registration systems in low- and middle-income countries consistently reveal substantial weaknesses in the generation of cause-of-death statistics.^9^^–^^11^ In a recent editorial, we drew attention to the fact that even hospital statistics on cause of death cannot be assumed to be correct – a fact that is not widely appreciated by governments and other users of these data.^12^

The gold standard for cause-of-death reporting is to have the cause certified by a medical practitioner using the rules and procedures of the *International classification of diseases and related health problems* (ICD), which is currently available in its tenth revision (ICD-10).^13^ Although most countries with statistical systems for cause of death now use the ICD classification for coding, not all countries have introduced the international standard certificate for reporting cause of death. Furthermore, physicians often do not receive adequate training in standard ICD death certification practices. It is, therefore, not surprising that comparative assessments commonly find that the quality of medical certification of the cause of death is poor.^3^^,^^8^ Cause-of-death statistics of poor quality have limited policy utility and may even seriously mislead policy debates.^14^

In most developing countries, more than half of all deaths occur outside hospitals. Since out-of-hospital deaths are rarely medically certified, most of the physician-certified deaths come from hospitals.^3^^,^^4^ Can we automatically assume that the cause assigned to a death in hospital is accurate? Unfortunately, even in countries where hospital data are the only source of cause-of-death information, data quality is rarely evaluated. Research in different countries has repeatedly identified substantial misclassification of the cause of death of people who die in hospitals – with attendant implications for the use of cause-of-death data in informing policy.^14^^–^^20^

To carry out a validation study of cause-of-death data collected in hospitals, we need a gold standard against which the hospital cause-of-death reports can be compared. While autopsy findings provide the ideal gold standard for cause-of-death evaluations, this approach is prohibitively expensive, rarely applied and likely to be based on a biased sample of deaths assigned to coroners. It would not be practical to carry out autopsies for all of the deaths occurring in a country – or even for all of the hospital deaths in a country.^19^ Instead, researchers have reviewed the medical records of people who have died in hospitals as a reference standard for validating the accuracy of the causes of deaths recorded by the hospitals. Although all hospitals have medical records for their patients, such records are rarely used for carrying out routine assessments of the extent and nature of any diagnostic misclassifications among hospital deaths. In part, this reflects a lack of awareness of the existence of such misclassification or – because there is no standard method and framework for carrying out routine evaluations of the quality of cause-of-death data – a lack of knowledge of how such misclassification might be identified. Here, we perform a systematic review of studies that used medical records to assess the quality of hospital cause-of-death data to ascertain the pattern and extent of diagnostic misclassification of the cause of death. We propose a standard method for the future use of medical record reviews for assessing the accuracy of hospital-based cause-of-death data.

## Systematic review

### Search strategy

**Fig. 1:**
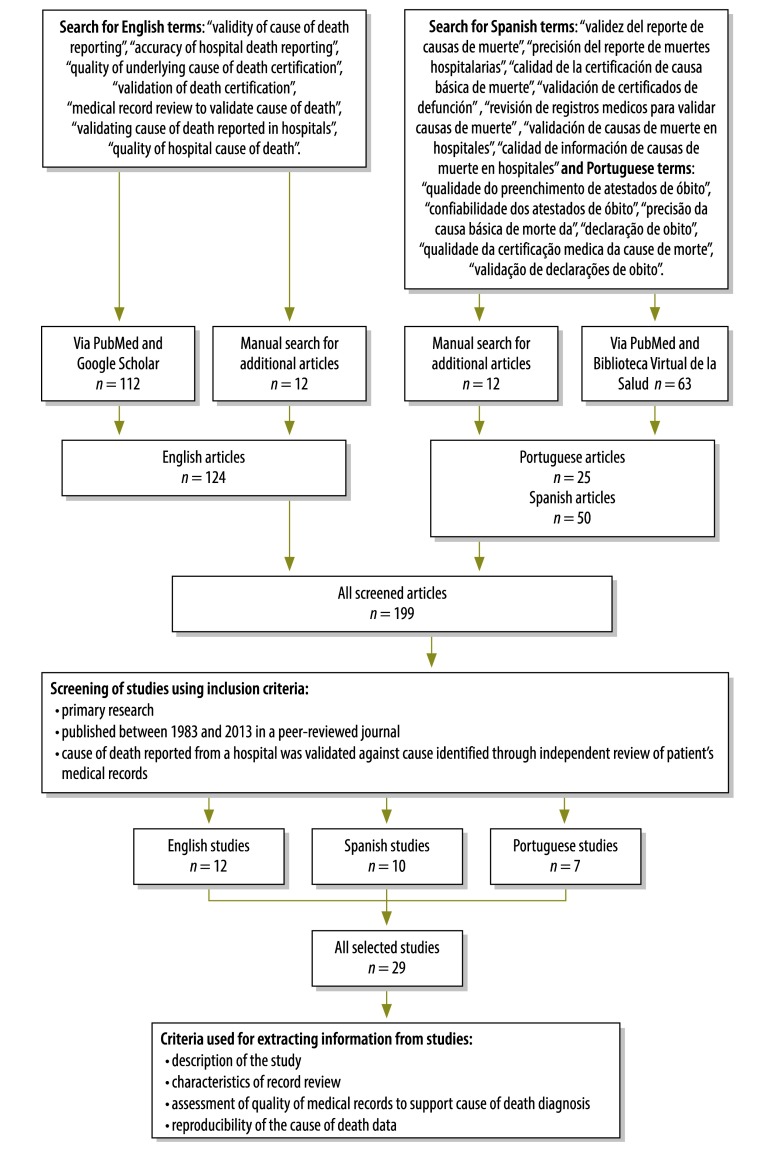
Flowchart for the selection of studies on medical record reviews to validate cause-of-death data for hospital deaths

We searched for published articles on studies that used medical record reviews to validate cause-of-death data for hospital deaths in English, Spanish or Portuguese, between 1983 and 2013. Search terms and databases are shown in (Fig. 1). All 199 studies identified from the initial investigation were screened for specific content and only the 29 studies that met our inclusion criteria were subjected to further scrutiny (Fig. 1, Appendix A available from: http://www.uq.edu.au/hishub/docs/Appendix%20A_final.pdf). Essentially, to be included in our systematic review, a study had to be primary research, published in a peer-reviewed journal and involve the validation of cause-of-death data originating from one or more hospitals against reference cause-of-death data obtained by review of the corresponding medical records.

## Results

The 29 selected studies were categorized according to timing, geographical location, scope and basic study features, age at death and range of causes of death included (Table 1). Wherever possible, we also assessed methodological issues such as the characteristics of the reviewers, the quality of the medical records used and the diagnostic facilities available in the study hospitals. Since the methods, scopes and objectives of the selected studies were diverse, no review protocol was systematically applied and no comparative analysis of our findings was possible.

**Table 1:** Key characteristics of the 29 studies selected for inclusion in the review^a^

Characteristic	Selected studies, no. (%)
**Year of study**	
After 2005^16^^,^^17^^,^^20^^–^^26^	9 (31.0)
2000–2004^14^^,^^18^^,^^27^^–^^32^	8 (27.6)
1990–1999^15^^,^^33^^–^^42^	11 (38.0)
Earlier than 1990^43^	1 (3.4)
**Location**	
Americas^15^^,^^16^^,^^21^^,^^24^^–^^26^^,^^29^^–^^34^^,^^37^^–^^42^	18 (62.1)
Europe^23^^,^^28^^,^^35^^,^^36^	4 (13.8)
Asia^17^^,^^18^^,^^22^	3 (10.3)
Pacific^20^	1 (3.4)
Africa^14^	1 (3.4)
Middle East^27^^,^^43^	2 (7.0)
**Sample size (no. of deaths)**	
0–99^15^^,^^40^	2 (6.9)
100–499^14^^,^^21^^,^^24^^,^^25^^,^^29^^–^^32^^,^^36^^–^^38^^,^^42^^,^^43^	13 (44.8)
500–1499^20^^,^^22^^,^^26^^–^^28^^,^^33^^,^^35^^,^^39^^,^^41^	9 (31.0)
More than 1500^16^^–^^18^^,^^23^^,^^34^	5 (17.3)
**Scope of the study**	
All conditions^14^^–^^18^^,^^20^^,^^22^^,^^24^^–^^26^^,^^28^^,^^30^^,^^34^^,^^35^^,^^38^^,^^39^^,^^42^^,^^43^	18 (62.2)
Cardiovascular conditions and/or diabetes^29^^,^^32^^,^^37^	3 (10.3)
All non-accidental deaths^21^	1 (3.4)
Ill-defined and vague causes^27^	1 (3.4)
Deaths with legal implications^23^^,^^36^	2 (7.0)
Neonatal causes^33^	1 (3.4)
Cancer^31^^,^^40^^,^^41^	3 (10.3)
**Age groups included in the study**	
All^14^^–^^18^^,^^20^^,^^22^^–^^27^^,^^31^^,^^32^^,^^35^^–^^43^	24 (82.8)
Adults^b,^^21^^,^^30^	2 (7.0)
Elderly^b,^^28^	1 (3.4)
Infants aged < 1 year^34^	1 (3.4)
Neonates^b,^^33^	1 (3.4)

^a^ Summary of the articles is available in Appendix A (Available from: http://www.uq.edu.au/hishub/docs/Appendix%20A_final.pdf).

^b^ Ages not defined in relevant article or articles.

Only nine of the 29 selected studies were published after 2005. The number of deaths included in each study ranged from 23 in a study in Mexico^15^ to 3316 in a nationally representative study of cause-of-death accuracy in Thai hospitals.^17^ More than half of the selected studies (*n* = 18) were carried out in the Americas; Mexico contributed three studies and Brazil six. There was only one study from the Pacific – in Tonga^20^ – and one from Africa – in South Africa.^14^ Eighteen studies considered all causes of death while the remainder (*n* = 11) investigated selected causes only. The large majority (*n* = 24) covered all age groups. However, two studies included only adult deaths, one study focused on deaths in the elderly and another two studies focused on infant deaths (Table 1).

The main purpose of most of the medical record reviews was to identify the degree of misclassification of cause of death at the individual level – by comparing the hospital or vital registration diagnosis with a reference diagnosis based on a review of the dead patient’s medical records. A misclassification matrix is the primary outcome of several studies.^3^^,^^16^^–^^18^^,^^20^^,^^22^^,^^27^ An example of a misclassification matrix, based on empirical research carried out in Colombo, Sri Lanka,^22^ is shown in Table 2. Selected findings from the misclassification matrices obtained in different countries are presented in Box 1. The misclassification matrix produced in a study that is nationally representative can be used to derive a series of correction factors that can be applied to routine cause-of-death data – from vital registration systems – to estimate the probable true cause-of-death pattern in the study country. In Thailand, for example, cause-specific mortality fractions that had been corrected in this manner were applied to the numbers of registered deaths in 2005 – which had been adjusted for underreporting – to estimate the probable true pattern of causes of death in the country in that year.^44^ For some causes of death, such as human immunodeficiency virus infection/acquired immunodeficiency syndrome and ischaemic heart disease, the corrected numbers of deaths in this study were 3- to 4-fold higher than the numbers recorded in the vital registration system – with huge implications for Thailand’s health policies. Fifteen of our selected studies^16^^–^^18^^,^^20^^–^^23^^,^^27^^,^^28^^,^^31^^–^^33^^,^^38^^,^^39^^,^^44^ provided information to correct cause-specific mortality fractions based on vital registration data – where these fractions were known or suspected to be incorrect.

**Table 2:** Misclassification of causes of deaths, all ages, both sexes combined, Colombo, Sri Lanka, 2012

Vital registration diagnosis	Diagnosis based on medical records review (no. of deaths)																	
	Certain infectious and parasitic diseases	All cancers	Diabetes mellitus	Other diseases of the nervous system	Hypertensive diseases	Ischaemic heart diseases	Cerebrovascular diseases	Other heart diseases	Pneumonia	Chronic lower respiratory diseases	Other diseases of the respiratory system	Diseases of the liver	Diseases of the skin	External causes	All other causes		Total	
Certain infectious and parasitic diseases	9	3	1	0	0	0	0	2	0	1	0	1	0	2	4		23	
All cancers	1	34	1	0	0	2	3	0	0	4	0	2	0	0	3		50	
Diabetes mellitus	3	3	34	1	4	22	9	1	3	2	0	3	1	1	7		94	
Other diseases of the nervous system	2	0	0	3	0	2	0	0	1	0	1	2	0	0	0		11	
Hypertensive diseases	4	0	2	0	12	9	10	0	1	3	0	1	0	0	2		44	
Ischaemic heart diseases	2	0	9	0	2	54	5	3	1	5	0	4	0	1	3		89	
Cerebrovascular diseases	0	1	1	1	2	1	17	1	0	1	1	1	0	0	1		28	
Other heart diseases	1	4	3	0	2	17	4	21	1	4	1	3	1	0	8		70	
Pneumonia	0	1	1	2	0	1	1	0	9	2	0	0	0	0	6		23	
Chronic lower respiratory diseases	1	1	1	0	0	7	1	1	1	13	0	0	0	0	2		28	
Other diseases of the respiratory system	0	1	1	1	0	1	1	0	1	0	3	2	0	1	3		15	
Diseases of the liver	4	2	2	1	2	2	0	3	0	1	0	39	1		3		60	
Diseases of the skin	0	1	0	0	0	1	0	0	0	0	0	1	0		1		4	
External causes	3	3	3	0	2	3	0	1	0	1	0	2	0	1	5		24	
All other causes	2	2	3	2	1	5	3	2	1	1	0	3	0	0	14		39	
Total	32	56	62	11	27	127	54	35	19	38	6	64	3	6		62		602

Data source: Based on data collected by Rampatige et al.^22^

Box 1 **Selected findings based on reported misclassification matrices for causes of hospital deaths in four countries**

**China**

Rao et al.^18^ have shown that, ischaemic heart disease was undercounted in the official statistics by 31% because of the systematic misclassification of true cases of ischaemic heart disease to stroke, diabetes, pneumonia or other forms of heart disease. Hepatitis deaths were found to be frequently misclassified to other liver diseases, and pneumonia was found to be excessively and often incorrectly selected, from a list of respiratory diseases, as the underlying cause of death.

**Islamic Republic of Iran**

Khosravi et al.^27^ have found that the true cause-of-death pattern of the population was found to be considerably different from the pattern of causes reported by the vital registration system in the country. The ill-defined causes reported by the routine death registration system for many deaths among young and middle-aged adults were primarily reclassified, after review, to ischaemic heart disease, stroke and injuries. In half of the study sample, injury deaths had been classified as senility or unknown in the vital registration system – thus greatly underestimating the importance of external causes of hospital deaths. Ill-defined causes of death at an age of ≥  70 years were largely reclassified, after review, to ischaemic heart disease and stroke.

**Sri Lanka**

Rampatige et al.^22^ have revealed major misclassification errors in identifying deaths caused by vascular diseases or diabetes. Of the deaths caused by ischaemic heart disease, 30% had been misclassified to diabetes or another heart disease and 25% of the deaths due to diabetes mellitus had been misclassified as various diseases of the circulatory system.

**Thailand**

Pattaraarchachai et al.^17^ also reported massive misclassification of major causes of death. Cases of septicaemia – commonly reported in the vital registration system – were reassigned to cerebrovascular disease, human immunodeficiency virus infection/acquired immunodeficiency syndrome and pneumonia. Ill-defined causes were identified as true cases of ischaemic heart disease, other heart disease, chronic obstructive pulmonary disease or stroke. The study also found gross underdiagnosis of diabetes by the vital registration system.

The utility of any medical record review depends on the ability of the reviewers to identify the underlying cause of death correctly from the medical records.^45^ The reviewers in most of our selected studies (*n* = 18) were physicians who had – reportedly – been trained in death certification practices (*n* = 15). One study simply reported that professionals had been used as reviewers, while nine studies provided no information about reviewer qualifications. A Brazilian study^34^ that used both a physician and a researcher was classified as physician-review in this analysis. The number of reviewers per study depended on sample size and ranged from one in each of four studies to 84 in the Thai study.^17^

The accuracy of the reference cause-of-death diagnosis depends on the quality of information available in the medical records. Ten of the reviewed studies included such an assessment – in which the records were categorized either simply, as adequate or inadequate, or, with more qualification, as excellent, good, average, weak or poor. One study excluded deaths when the corresponding medical records were judged to be incomplete.^23^ The remainder provided no information about the quality of the medical records used.

In nine of our selected studies, standard diagnostic criteria were pre-determined – i.e. the clinical evidence required to classify a death as being due to a particular cause was specified in advance of the review.^14^^,^^16^^,^^20^^,^^23^^,^^25^^,^^38^^,^^40^^,^^41^^,^^43^ In case of diagnostic uncertainty, nine studies used a physician panel to discuss diagnostic problems. Three studies ordered verbal autopsies when the cause of death was in doubt. One study referred diagnostic problems to a second physician, and two others referred to external experts. Fourteen studies provided no information about the resolution of diagnostic uncertainty.

The diagnostic capacity of hospitals will clearly influence the accuracy of their cause-of-death diagnoses, both in vital registration systems and in medical record reviews. Only the Chinese study^18^ specifically mentioned that urban hospitals were selected to ensure that the study hospitals had adequate diagnostic facilities. None of the other studies included in our analysis referred to hospital diagnostic capacity.

The correct assignment of the underlying cause of death on the death certificate requires not only that the sequence and relationship of pathological conditions on the certificate are correctly certified but also that the underlying cause of death is correctly coded in terms of the ICD. Despite the critical role of coding, only three studies assessed coding accuracy.^17^^,^^26^^,^^37^

All studies reported some degree of diagnostic misclassification. Due to the use of different classification lists, the misclassification reported in these studies are not directly comparable. For the studies that have used ICD-10’s three-character codes for comparison of cause of death, the range of misclassification varied from 25^24^ to 62%.^16^ The concordance at the level of ICD-10’s three-character codes was 41.4% in Sri Lanka^22^ while concordance at the level of the Basic Tabulation List in the ninth revision of the ICD was found to be 77% in Sweden.^35^ In South Africa, concordance at the level of ICD-10’s mortality tabulation level was 55.3%.^14^ The pattern and extent of misclassification found in the studies were generally reported either as matrices or as percentages. Other metrics that were used included simple concordance-based measures of sensitivity and specificity, and measures that attempt to correct for the probability of getting a concordant diagnosis merely by chance, such as Kappa statistics and chance-corrected concordance.^46^ More usefully, the analysis of misclassification matrices allows specific insight into the patterns of cause-of-death misallocation and is thus a critical input in activities designed to strengthen health information systems as well as in the correct interpretation of national mortality statistics. Common causes of death that were frequently misclassified included ischaemic heart disease, stroke, diabetes mellitus and external causes of injury. In a study based on a medical record review in the Islamic Republic of Iran, ill-defined causes of death were mainly reclassified to ischaemic heart disease (33.5%) and cerebrovascular accidents (17.1%)^27^ while a study in Kuwait reported that original death certificates underestimated cerebrovascular disease by 69%, diabetes mellitus by 60% and ischaemic heart disease by 33%.^43^ Surprisingly, in our selected studies, ill-defined causes of death were frequently reported even in hospital settings. The policy impact of allocating these non-specific codes to more definitive causes are reported in two studies;^21^^,^^44^ key findings from a meta-analysis of misclassification patterns can be found in the article by Rampatige et al.^47^

### Framework for reviewing medical records

Given the importance and cost of accurate cause-of-death statistics for health policy and priority setting – and the effort and cost of undertaking medical record reviews – evaluation studies using this method could benefit enormously from some simple, evidence-based lessons. Based on this systematic review and experience with our own field research, we provide a synthesis of some of the key issues that should guide national applications of medical record reviews.^16^^–^^18^^,^^20^^,^^22^^,^^27^

Our systematic review confirms that a variety of approaches have been used to assess the accuracy of cause-of-death data. Rather than trying to evaluate whether there is one approach that is better than the others, we have tried to draw lessons from each study about the specific steps that added value to the study and could be repeated elsewhere. Building on these findings, we propose a framework that addresses the critical choices that need to be carefully considered when using medical record reviews for assessing cause-of-death data quality (Box 2). Further detail on each of the topics included in our framework has been published elsewhere – with specific guidance on the selection of hospitals and cases, the development and use of diagnostic criteria, interpreting misclassification matrices, evaluating the quality of the source documentation, and the choice and training of the reviewers used.^47^

Box 2 **Proposed framework and key elements for conducting medical record reviews**

**Select hospitals to be reviewed**

- Determine scope of investigation
- Obtain agreement for hospital cooperation
- Conduct census of diagnostic facilities available in included hospitals.

**Select diagnostic categories and develop diagnostic criteria**

- Set up a small expert group of physicians to develop standard diagnostic criteria
- Establish a list of the diseases that are the most important for the review
- Develop and pre-test diagnostic criteria on a sample of medical records.

**Select sample death certificates**

- Determine sample size
- Determine the sampling method and identify the number of death certificates to be included in the study
- Draw the sample of death certificates from the vital registration database or hospital mortality register
- Retrieve corresponding medical records from the hospitals
- Validate the quality of coding – according to the tenth revision of the *International Classification of Diseases and Related Health Problems* (ICD-10) – for the sample.

**Select physicians to rediagnose causes of death**

- Provide training in cause of death certification.

**Trace the relevant medical records**

- Decide on criteria to assess the quality of the records
- Decide on rules to determine which records can be used and which are too incomplete
- Reassess the sample size because of losses arising from poor or untraceable records
- Prepare a summary of the quality and availability of medical records and the corresponding maintenance practices.

**Review medical records**

- Design form for extracting information from medical records. An example of such medical data extraction forms is given in the study by Rampatige et al.^47^
- Diagnose cause of death using pre-defined standard diagnostic criteria
- Code the new cause according to ICD-10
- Check that coding is correct.

**Compare the cause of death originally reported from vital registration with that finally assigned after medical record review, and analyse findings**

- Determine the extent of misclassification
- Draw up a misclassification matrix for all ages and both sexes – and by age and sex, if numbers allow – to identify patterns of misclassification
- Reassign the ill-defined and misclassified causes based on the misclassification matrix
- Compare the new distribution of causes of death with the original, to identify major discrepancies.

**Write final report and initiate policy dialogue to strengthen health information system**

- Describe the study design and methodology, including sampling strategy
- Discuss findings and implications
- Propose improvement steps as needed – e.g. improved certification, coding and keeping of medical records.

## Discussion

Medical record reviews can and have been carried out to serve many different purposes. Perhaps the most common application is the independent assessment of the reliability of hospital cause-of-death data – particularly when expert review or other use of mortality data has revealed these to be deficient. Another common purpose is to assess whether deaths from specific causes – e.g. various cancers or traffic accidents – are being reliably recorded in hospital settings. If such an assessment involves a reasonably representative national sample of hospital deaths, correction factors can be calculated from the misclassification matrices and applied to national cause-of-death data to estimate the likely true cause-specific mortality pattern in the population.

Despite an extensive literature review, we were only able to identify a small number of peer-reviewed studies that had used medical records to investigate the quality of cause-of-death data (*n* = 29). Given the critical role of cause-of-death data in informing debates around national health policies, this is both surprising and disappointing. Our main observation – of extensive misclassification of causes of death in hospitals wherever studies have been carried out – indicates that inaccurate cause-of-death data are pervasive. However, it is important to consider potential biases that might have affected the limited number of studies that we were able to analyse. First, we were only able to review articles that had been published in a language in which at least one member of our review team was competent – i.e. English, Portuguese or Spanish. It is possible that articles published in other languages might have led to different conclusions about the pattern and extent of cause-of-death misclassification in hospitals. More comprehensive studies covering other major language groups would be welcome but there is no reason to suspect that these would not reinforce our main findings – which seemed fairly consistent irrespective of the language of the article involved. Thus, our conclusions and recommendations for policy action should still be valid. It is also possible that the literature we screened was affected by selective biases – such as publication bias – but we suspect that this would only operate to suppress the publication of more extreme findings about misclassification. Finally, the studies we selected had diverse aims and the set of causes of death investigated in each study was presumably biased towards the priorities of the interest groups involved. We found only one study that involved the comprehensive investigation of cause-of-death misclassification across all major causes of death. It is therefore quite likely that the extent of cause-specific misclassification across all countries is different to that indicated by the studies we selected. Again, however, that does not alter our fundamental conclusion that hospital statistics on causes of death – whatever the universe of causes investigated – are likely to be grossly inaccurate in many countries and ought to be the focus of an immediate policy response.

All of the studies we selected for review assessed the quality of the hospital-reported cause-of-death data by comparing them with cause-of-death data derived by reviewing medical records. However, the lack of a standard framework or method for conducting and reporting the findings of medical record reviews has resulted in substantial variation in the approaches used and difficulties in interpreting and comparing results. Building on these findings and our own empirical experience, we propose a series of steps to guide future studies on the accuracy of cause-of-death data that involve the review of medical records. By following these steps, it should be possible to validate the quality of cause-of-death data reported by hospitals and vital registration systems more reliably.

The synthesis of lessons learnt, summarized in Box 2, outlines a clear and tested method and identifies the most important steps in the process – e.g. the design of the study, development of diagnostic criteria, selection of the sample, review of medical records, and the analysis and interpretation of findings. However, the framework that we have proposed – like all frameworks – has limitations. In particular, it would not be feasible to apply our framework in settings where medical records are poorly kept or where the diagnostic capacity of hospitals is generally poor. An understanding – by hospital and health administrators – of the importance of studies based on our framework for policy-makers is crucial, as is a commitment, of those responsible for the development of national health information systems, to implement any resultant recommendations.

While the proposed framework needs to be tested in its entirety in further empirical studies, we consider it sufficiently robust to be applied in its current state in different settings. We recommend its periodic application in all countries, to identify deficiencies in national mortality statistics. Using the misclassification matrix that the study method yields, health authorities should be able to identify the diseases that are commonly misclassified as causes of death and also ascertain whether the causes of death that are being recorded in hospitals are of sufficient quality to be fit for purpose. This knowledge can and should be used to guide strategies for the strengthening of health information systems – e.g. increasing awareness among medical associations about the value of doctors correctly certifying deaths, improving the keeping of medical records in hospitals, and ensuring that cause-of-death coders are appropriately trained.

Systems for the determination and recording of the causes of hospital deaths represent a purposeful and costly investment that countries make. It is imperative that such systems perform to the standard required to support good public policy.
